# Comparative study on the age-related incidence of seborrheic keratosis and verruca plana in patients with verruca plana-like lesions

**DOI:** 10.1038/s41598-024-55617-1

**Published:** 2024-03-04

**Authors:** Han-Seul Kim, So Yeon Myeong, Hee Young Kang, Jin Cheol Kim

**Affiliations:** 1 Department of Dermatology, Ajou University School of Medicine, Suwon, South Korea; 2 Department of Biomedical Sciences, Molecular Medicine, Graduate School of Ajou University, Suwon, South Korea

**Keywords:** Age factors, Aged, Keratosis, Seborrheic, Prevalence, Skin neoplasms/etiology, Warts, Skin manifestations, Skin diseases, Epidemiology

## Abstract

Seborrheic keratosis (SK) is a common skin disease in the elderly. However, in cases where SK presenting as multiple skin-colored or clustered lesions can be easily misdiagnosed as verruca plana (VP), especially in the young population. This retrospective study investigated the prevalence of SK and VP in the lesions that appear clinically similar to VP according to age. We examined the pathology slides of the skin tissue and photographs of patients who were clinically suspected to have VP. A total of 503 patients were included in the study, out of which 174 patients were finally diagnosed with SK (34.6%) and 132 with VP (26.2%). The mean ages of the SK- and VP-diagnosed group were 39.3 and 35.4 years, respectively. SK had a higher prevalence among individuals older than 30 years, and relative frequency of SK should not be ignored in patients with a grouped distribution in their 20 s and 30 s. Therefore, our study suggests that multiple verrucous skin-colored to brownish plaques are also commonly diagnosed as SK in young people as well as VP, and the prevalence of SK and VP may not always depend solely on chronological aging, and the prevalence of SK among young people may be higher than commonly believed stereotypes suggest.

## Introduction

Seborrheic keratosis (SK) is one of the most common benign skin lesions with a higher prevalence in the elderly^1^. In contrast, verruca plana (VP) is an infectious skin disease caused by human papillomavirus and more commonly observed in children and early adulthood^2^. VP presents as multiple small papules on the face and dorsum of the hands and causes major cosmetic concerns in patients^2^. In addition, VP is highly contagious and is usually transmitted through direct contact^2,3^; thus, it can cause substantial psychological distress in patients, especially among those around immunosuppressed patients, pregnant women, or children in their families. Unlike typical SK that presents as slightly raised brownish plaques, some SKs share similar morphological characteristics with VP such as multiple small-sized skin-colored papules^4–6^. Because of such similarities between SK and VP, dermatologists often experience difficulties in clinically differentiating between the two diseases^5,7–9^. Therefore, a skin biopsy is necessary to diagnose VP and SK distinctly, and empirical treatments should be considered for SK in old age and VP in young age^7,9–11^. However, recent evidence suggests that SKs occur in young people as well^12–14^. Up to 24% of the general population in Australia, aged between 15 and 30 years, were reported to have SK^12^. In the UK population, 17% of women under the age of 40 years had at least one form of SK^13^. Thus, in this study, we analyzed the histology of clinically diagnosed VP-like lesions according to age in a real-world setting, assessed the prevalence of SK that clinically mimics VP in young adults between SK- and VP-diagnosed patients.

## Results

### Demographics and clinicopathological characteristics of study population

**Figure 1:**
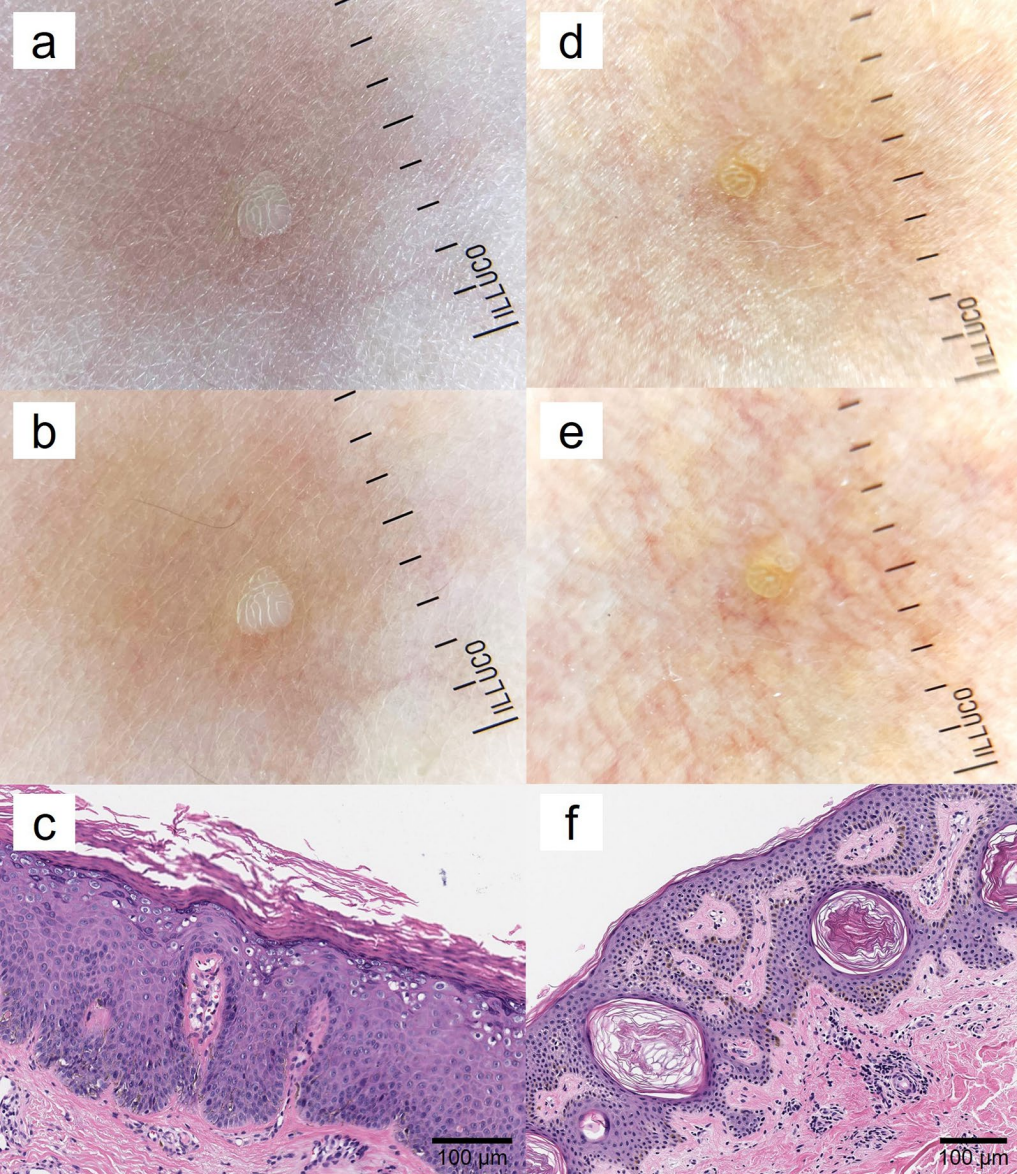
Discrepancy between dermoscopic findings and histopathological results of verruca plana (VP)-like seborrheic keratosis (SK) and verruca plana. (**a**, **b**) Dermoscopic finding shows brain-like appearance suggesting a higher likelihood of SK. (**c**) Histopathological examination of the lesions with features highly indicative of SK (**a**, **b**) revealed numerous koilocytes, ultimately confirming a diagnosis of VP. (**d**, **e**) Dermoscopic finding shows also brain-like appearance suggesting a higher likelihood of SK. (**f**) Histopathological examination of the lesions with features highly indicative of SK (**d**, **e**) revealed epidermal acanthosis and horn pearls, finally confirmed with SK.

**Figure 2:**
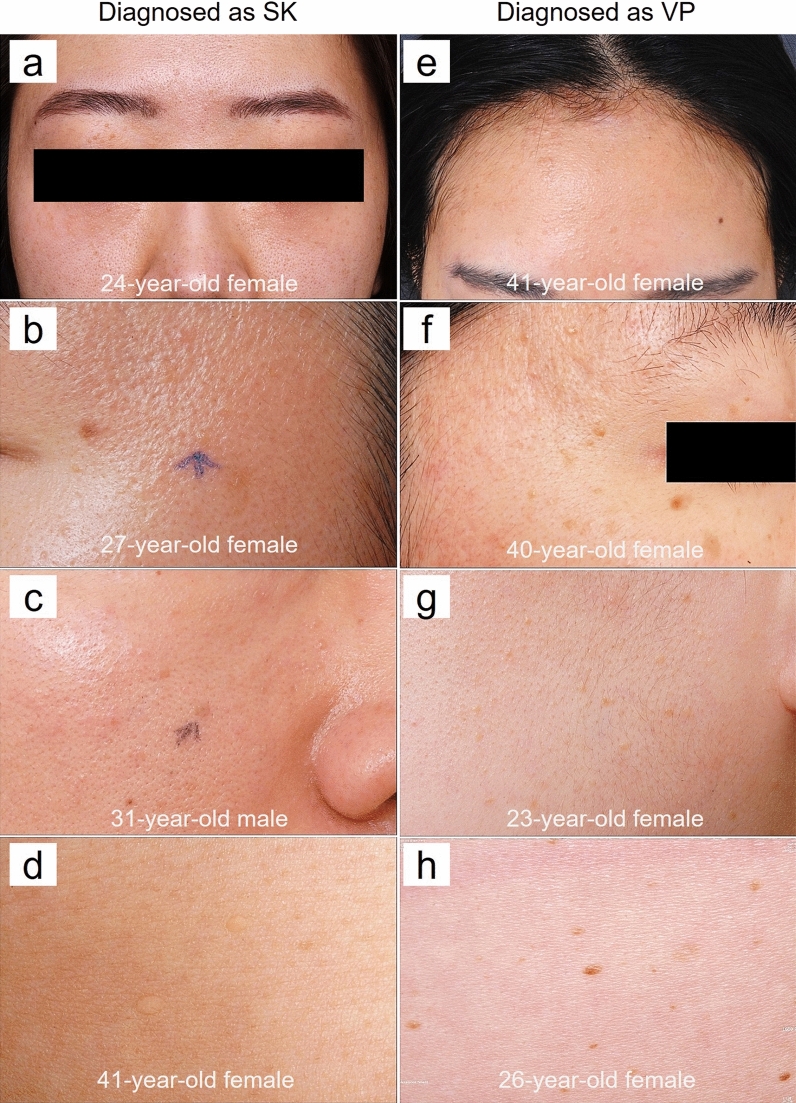
Representative clinical photographs of verruca plana (VP)-like seborrheic keratosis (SK) and verruca plana in different age groups. (**a**) Multiple skin-colored papules on the face that diagnosed as SK. (**b**, **c**) Skin-colored to brownish papules on face that diagnosed as SK. (**d**) Skin-colored round papules on arm that diagnosed as SK. (**e**) Multiple skin-colored papules on the face that diagnosed as VP. (**f**, **g**) Skin-colored to brownish papules on face that diagnosed as VP. (**h**) Brownish papules on trunk that diagnosed as VP.

Most VP-like lesions were diagnosed with SK or VP (Figs. 1, 2); 174 cases were diagnosed with SK (34.6%) and 132 with VP (26.2%) (Table 1). Other common diagnoses were syringoma (6.2%), milium (3.4%), folliculitis (3.2%), chronic dermatitis (2.6%), and sebaceous hyperplasia (2.2%). The pathological diagnoses for all cases are shown in Supplementary Table 1. There were 330 females (75.2%), and the mean age at the time of biopsy was 37.7 years (range 1–88 years). Most lesions were located on the face (48.5%), followed by the extremities (25.4%). The number of total lesions was generally more 5 but less than 30 (53.3%). In most cases, the lesion size was < 3 mm (61.6%). A majority of VP-like lesions (283 cases) represented skin-colored lesions (56.3%), while the remaining appeared brown (43.7%). A total of 293 patients (58.3%) showed a grouped distribution. Most patients diagnosed with SK were females (80.5%), had lesions on the face (52.9%); the lesions were > 5 (89.6%), were small than 3 mm (70.1%), brown-colored (65.5%), and were round in shape (83.9%). In contrast, 53.0% of the patients diagnosed with VP were female; among them, the lesions were usually located on the face (43.2%), followed by the extremities (39.4%). The lesions in patients diagnosed with VP were commonly larger than 3 mm, (52.3%), heterogeneous in size (57.6%), skin-colored (65.2%), round (75.8%), and grouped (68.2%). Patient sex (P < 0.001), lesion location (P < 0.001), size (P < 0.001), color (P < 0.001), and distribution (P < 0.001) were significant independent predictors for the histological diagnosis of SK. However, there were no statistically significant differences between the SK and VP groups with respect to the number of lesions and heterogeneity of lesion size, shape, or height.

**Table 1:** Demographics of the study population.

	Total	SK	VP	*P*-value
Number of patients, N (%)	503 (100.0%)	174 (34.6%)	132 (26.2%)	
Sex, N (%)				< 0.001
Female	330 (65.6%)	140 (80.5%)	70 (53.0%)	
Male	173 (34.4%)	34 (19.5%)	62 (47.0%)	
Age, mean (standard deviation)	37.7 (15.0)	39.3(11.4)	35.4 (14.2)	
Age distribution, N (%)				0.001
< 10	17 (3.4%)	0 (0.0%)	5 (3.8%)	
10–19	33 (6.6%)	2 (1.1%)	12 (9.1%)	
20–29	80 (15.9%)	24 (13.8%)	26 (19.7%)	
30–39	161 (32.0%)	79 (45.4%)	42 (31.8%)	
40–49	98 (19.5%)	32 (18.4%)	24 (18.2%)	
50–59	84 (16.7%)	28 (16.1%)	17 (12.9%)	
60–69	22 (4.4%)	6 (3.4%)	5 (3.8%)	
70–79	7 (1.4%)	3 (1.7%)	1 (0.8%)	
80–89	1 (0.2%)	0 (0.0%)	0 (0.0%)	
Site of lesions, N (%)				< 0.001
Face	244 (48.5%)	92 (52.9%)	57 (43.2%)	
Neck	37 (7.4%)	13 (7.5%)	11 (8.3%)	
Scalp	3 (0.6%)	1 (0.6%)	0 (0.0%)	
Trunk	91 (18.1%)	49 (28.2%)	12 (9.1%)	
Extremity	128 (25.4%)	19 (10.9%)	52 (39.4%)	
Number of lesions, N (%)				0.771
≤ 5	66 (13.1%)	18 (10.3%)	17 (12.9%)	
5–30	268 (53.3%)	105 (60.3%)	76 (57.6%)	
≥ 30	169 (33.6%)	51 (29.3%)	39 (29.5%)	
Size of lesions, N (%)				< 0.001
Small (< 3 mm)	310 (61.6%)	122 (70.1%)	63 (47.7%)	
Large (≥ 3 mm)	193 (38.4%)	52 (29.9%)	69 (52.3%)	
Heterogeneous size, N (%)	260 (51.7%)	85 (48.9%)	76 (57.6%)	0.130
Color, N (%)				< 0.001
Skin colored	283 (56.3%)	60 (34.5%)	86 (65.2%)	
Brown	220 (43.7%)	114 (65.5%)	46 (34.8%)	
Shape, N (%)				0.075
Round	403 (80.1%)	146 (83.9%)	100 (75.8%)	
Irregular	100 (19.9%)	28 (16.1%)	32 (24.2%)	
Height, N (%)				0.059
Papuloplaque	474 (94.2%)	164 (94.3%)	130 (98.5%)	
Maculopatch	29 (5.8%)	10 (5.7%)	2 (1.5%)	
Distribution, N (%)				< 0.001
Scattered	210 (41.7%)	90 (51.7%)	42 (31.8%)	
Grouped	293 (58.3%)	84 (48.3%)	90 (68.2%)	

*SK, seborrheic keratosis; VP, verruca plana.

### Prevalence of SK and VP in VP-like lesions according to chronological age

**Figure 3:**
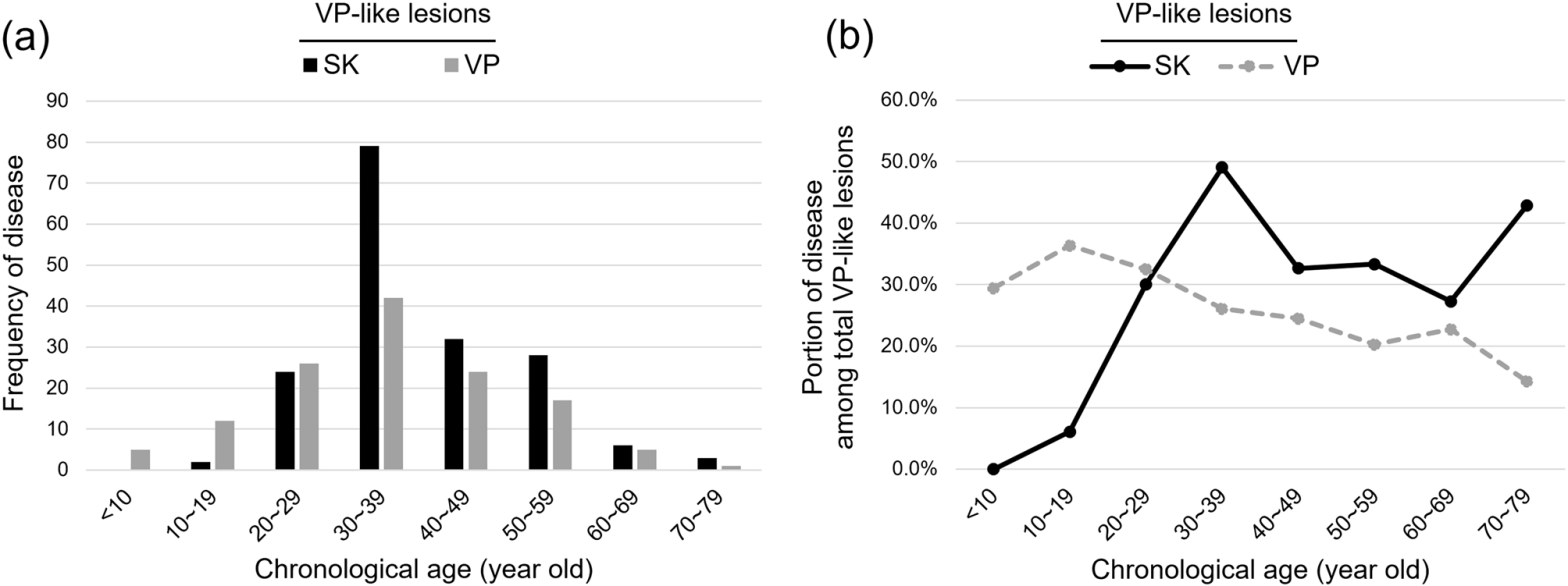
Patients aged > 30 years with highly suspicious verruca plana (VP)-like lesions are more likely to be diagnosed as having seborrheic keratosis (SK). (**a**) Comparison of frequency of SK and VP according to chronological age distribution in the study population. (**b**) Comparison of prevalence of SK and VP among all categories of VP-like lesions according to chronological age distribution.

The age distribution of cases pathologically diagnosed with SK or VP in VP-like lesions are presented in Table 1 and Fig. 3. The mean age of patients pathologically diagnosed with SK was 39.3 years, and that of those with VP was 35.4 years. The age group with the highest prevalence of VP-like lesions was in the range of 30–39 years, and the frequency of SK in this age range was higher than that of VP (57.2% and 30.4%, respectively). Patients aged < 30 years had a higher prevalence of VP (29.4% for 0–9 years; 40.0% for 10–19 years, and 35.1% for 20–29 years), whereas SK occurred more frequently in patients aged > 30 years (40.0% for 40–49 years; 39.4% for 50–59 years, 28.6% for 60–69 years, and 42.9% for 70–79 years).

**Figure 4:**
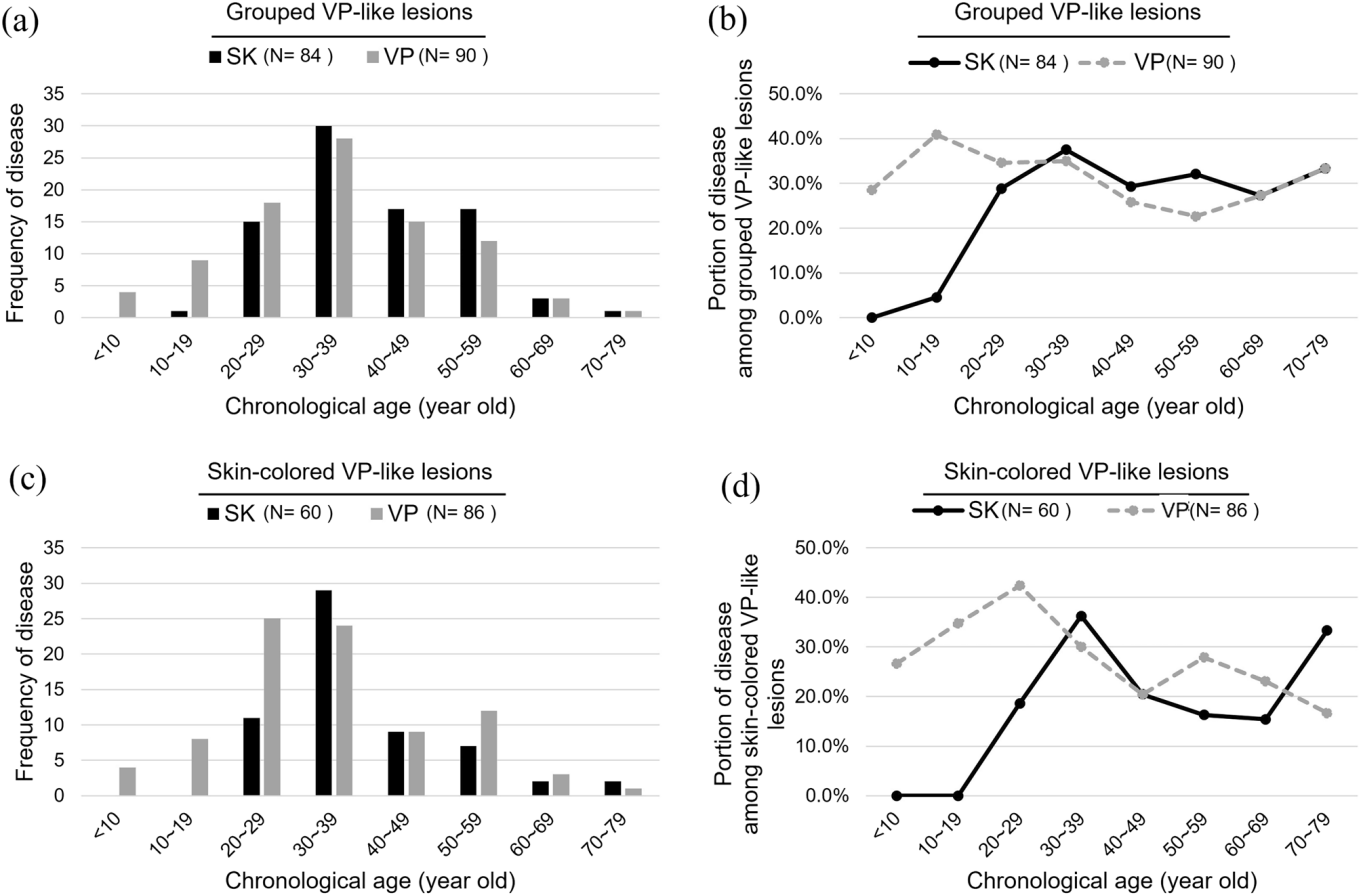
Subgroup analysis of age distribution and prevalence of verruca plana (VP) and seborrheic keratosis (SK) in the subgroup with grouped or erythematous to skin-colored lesion. (**a**) Age distribution of VP and SK with grouped lesions. (**b**) Prevalence of VP and SK in grouped lesions subgroup. (**c**) Age distribution of VP and SK with erythematous to skin-colored lesions. (**d**) Prevalence of VP and SK in erythematous to skin-colored lesions subgroup.

In cases with a grouped distribution, patients aged 30–59 years were more frequently diagnosed with SK (Fig. 4A,B, 37.5% for 30–39 years; 29.3% for 40–49 years; 32.1% for 50–59 years). Regarding skin-colored lesions, patients aged 30–39 and 70–79 years had a higher prevalence of SK (Fig. 4C,D, 36.3% for 30–39 years; 33.3% for 70–79 years).

## Discussion

In our study, among 503 patients with VP-like lesions, 34.6% were diagnosed with SK, whereas 26.2% were diagnosed with VP, based on histopathological results. If the patient is female, or if the lesion is on the face, less than 3 mm in size, has a brownish color, and is distributed in a scattered pattern, the likelihood of diagnosis of SK increases. Among patients with SK and VP, the average ages at the time of diagnosis were 39.3 and 35.4 years, respectively. In addition, the 30–39 age group showed a higher proportion of SK-diagnosed cases than VP-diagnosed cases. Both SK-diagnosed- and VP-diagnosed cases showed a similar prevalence in the 20–29 age group. These findings suggested that clinically presented VP-like lesions were mostly SK, and the prevalence proportion of SK should not be ignored in patients in their 1920s and 1930s.

SK mainly affects the elderly according to previous literature. According to some studies that have revealed the prevalence of SK, the mean age of patients with SKs has increased over time^15,16^. Roh et al.^16^ investigated 206 Korean patients with SK who were diagnosed with biopsy-proven SK and found that the mean age among patients was 60.9 years. However, in a previous study that investigated 170 Australian individuals aged 15–30 years, the prevalence of SK was 32.3% in the 25–30-year group^12^. They suggested that SK is also common in young people, and that the term senile keratosis, another term for SK, was no longer appropriate.

An attempt was made to clarify the mismatched clinical diagnosis that was pathologically proven as SK, and the results showed that verruca vulgaris and VP were common clinical misdiagnoses in 26 cases of biopsy-proven SK^16^. Since some non-typical SK lesions are characterized as slightly raised or flat-topped light brown to skin-colored papules, they could be clinically misdiagnosed as VP. Kwon et al. investigated 303 Korean male patients with SK and reported that SK on sunlight-exposed skin was usually smaller than that on partly exposed skin, especially in the younger age groups^17^. They also found that the mean number of SK papules was higher in the sunlight-exposed areas than in the partly exposed areas^17^. These may result in more confusion in determining the diagnosis of VP-like lesions in the young population, especially in sun-exposed areas such as the face and neck, as SK is commonly represented as a solitary and larger lesion than VP (Fig. 2).

Patients often experience anxiety related to the transmission or aesthetic outcomes of VP^7,18,19^. In a study on French patients that investigated the psychological consequences of warts, 47.6% of patients reported feeling moderately to extremely anxious or depressed^19^. Furthermore, although VP usually requires treatment because of high risk of transmission to others, SK does not cause medical problems other than cosmetic concerns. Therefore, differentiation between these two diseases is required to relieve the patients from unnecessary distress and for medical counseling.

Previous studies have investigated the clinical characteristics that can be helpful in differentiating these two diseases^7–9^. Kim et al.^8^ suggested that VP showed a more grouped or clustered distribution, red dots, or globular vessels in dermoscopic findings, and even-colored light brown to yellow patches than VP-like SK. Similarly, other studies have suggested that VP-like lesions that show a grouped distribution or a skin-like to pink color are more likely to indicate VP than VP-like SK^7^. However, in our study, even after distinguishing VP-like lesions for grouped distribution or erythematous to skin-like color, we revealed that differentiating VP from SK entirely based only on clinical findings could not be possible. Despite there have been several studies on clinical and dermoscopic findings to distinguish between SK and VP^6,7,9^, clinicians often encounter cases in the real world that are difficult to diagnose with only these findings (Fig. 1). Therefore, dermatologists should not hesitate to perform a skin biopsy in highly-suspected cases, in order to differentiate between these two conditions.

It has been known that senescent cells in skin are increasing with aging. Kim et al.^20^ showed that both fibroblasts and keratinocytes increased significantly with age. Also, there have been evidences suggesting that keratinocytes of SK lesions are in a senescent condition^21–23^. Thus, unexpected development of SK in young patients may be caused by accelerated cellular senescence, rather than their chronological age. Further studies are needed to identify the association with pathogenesis of SK and cellular senescence.

Our study had several limitations. The study population did not include all VP-like lesions. Also, there may is a potential risk of selection bias, as the included patients were those who underwent biopsies, possibly indicating the presence of atypical VP-like lesions. However, since we included all cases in which biopsy was performed with the clinical impression of both SK and VP, we believe that our results are still meaningful because this study enrolled all cases in which biopsy was necessary to confirm the diagnosis of VP-like lesions, owing to the difficulty in differentiating between the two diseases. In addition, the study was conducted through a retrospective review of clinical photographs and electronic medical records and included patients from a single tertiary center. Finally, due to the limitations of the retrospective study design, we were unable to investigate the patients' Fitzpatrick skin type. This limitation prevented us from accurately reflecting the patients' UV exposure or skin color.

In the present study, we observed that lesions clinically diagnosed as VP in young adults were also commonly diagnosed as SK. Despite the common conception that SK is diagnosed mostly in the elderly, we suggest that SK can occur in individuals of all age groups. As patients could suffer from unnecessary anxiety assuming the spread of the virus, we think our observations will be helpful in reassuring young patients with lesions that may be confused with VP, such as multiple, grouped, or skin-colored lesions.

## Materials and methods

### Data sources and study population

We retrospectively reviewed patients with VP-like lesions that were clinically diagnosed by a dermatologist and who underwent skin biopsy at Ajou University Hospital between January 2015 and May 2022. VP-like lesions were defined as “1–4 mm, slightly elevated, and flat-topped papules that have minimal scale”^2^. Tissue samples were histologically confirmed by a dermatopathologist, and clinical photographs of 503 patients were included. The medical records were examined for age and sex. The morphology and location of the lesions were assessed using clinical photographs. We classified the locations of these lesions as the face, neck, trunk, and extremities. The distribution of lesions was classified as grouped (> 5 lesions within 3.0 cm in diameter around the lesion) or scattered (only one lesion within 1 cm in diameter around the lesion or not meeting the criteria for grouped lesion) according to the dominant arrangement of lesions^9^. The lesions were also labelled as brown (including yellow) or skin colored (including pink). The number of lesions was categorized based on a value of 30, following findings from a previous study^7^. Similarly, the size of lesions was categorized using a threshold of 3 mm^7^. Heterogeneity of size was defined when less than 80 & of the lesion had equal size^8^. Among patients with VP-like lesions, we analyzed cases that were pathologically confirmed as SK or VP according to age. We also examined the prevalence of VP among those with a grouped distribution or erythematous to skin color, which are characteristics that suggest VP, as per previous studies^7,9^.

### Statistical analysis

Descriptive analyses, such as the chi-square test for categorical data, were performed to evaluate statistical significance. Results were considered statistically significant when the two-tailed p-value was less than 0.05. Statistical analyses were performed using SPSS Statistics 20.0. for Windows (IBM Corp. Armonk, NY).

### Ethical statement

The study design was reviewed and approved by the Institutional Review Board of the Ajou University Hospital (AJOUIRB-DB-2022-494). All experiments were performed in accordance with the relevant guidelines and regulations. Requirement for informed consent was waived by ethics committee of the Ajou University Hospital due to the retrospective nature of the study.

### Supplementary Information

Supplementary Table 1.

## Data Availability

The data that support the findings of this study are available on request from the corresponding author, JCK. The data are not publicly available due to their containing information that could compromise the privacy of research participants.
