# Supplementary material for: Comparative study on the age-related incidence of seborrheic keratosis and verruca plana in patients with verruca plana-like lesions
Source: Sci Rep. 2024 Mar 4;14:5223. doi: 10.1038/s41598-024-55617-1 (PMC10909870; doi:10.1038/s41598-024-55617-1)
Supplement: Supplementary file 1 — Supplementary Table 1. [file 41598_2024_55617_MOESM1_ESM.docx]

| **Supplementary Table 1. Diagnosis distribution in the study population** | |
| --- | --- |
| Diagnosis | N (%) |
| Seborrheic keratosis | 174 (34.6%) |
| Verruca plana | 132 (26.2%) |
| Syringoma | 31 (6.2%) |
| Milium | 17 (3.4%) |
| Folliculitis | 16 (3.2%) |
| Chronic dermatitis | 13 (2.6%) |
| Sebaceous hyperplasia | 11 (2.2%) |
| verruca | 8 (1.6%) |
| Actinic keratosis | 7 (1.4%) |
| Lentigo | 7 (1.4%) |
| Granuloma annulare | 6 (1.2%) |
| Lichenoid dermatitis | 6 (1.2%) |
| Lichen nitidus | 5 (1.0%) |
| Epidermal nevus | 3 (0.6%) |
| Lichen amyloidosis | 3 (0.6%) |
| Lichen planus | 3 (0.6%) |
| Lichen planus-like keratosis | 3 (0.6%) |
| Rosacea | 3 (0.6%) |
| Subacute dermatitis | 3 (0.6%) |
| Urticarial dermatitis | 3 (0.6%) |
| Fibrous papule | 2 (0.4%) |
| Interface dermatitis | 2 (0.4%) |
| Intradermal nevus | 2 (0.4%) |
| Junctional nevus | 2 (0.4%) |
| Molluscum contagiosum | 2 (0.4%) |
| Porokeratosis | 2 (0.4%) |
| Postinflammatory hyperpigmentation | 2 (0.4%) |
| Spongiotic dermatitis | 2 (0.4%) |
| Subacute eczematous dermatitis | 2 (0.4%) |
| Vellus hair cyst | 2 (0.4%) |
| Xanthoma | 2 (0.4%) |
| Etc.* | 27 (5.4%) |
|  |  |
| *Etc. contains angiolipoma, calcinosis cutis, compound nevus, dermatofibroma, fibroma, lymphangioma | |
